# Selecting the Next Class: The “Virtual Orthopaedic Rotation”

**DOI:** 10.5435/JAAOSGlobal-D-21-00151

**Published:** 2022-01-19

**Authors:** Joseph L. Yellin, Laura Y. Lu, Andrea S. Bauer, Jennifer Duane, Paul T. Appleton, Eric M. Berkson, Eric M. Bluman, Christopher M. Bono, Jacob M. Drew, Kaitlin Duffy, Harold A. Fogel, Collin May, John E. Ready, Michael J. Weaver, Bertram Zarins, George S.M. Dyer

**Affiliations:** From the Department of Orthopaedic Surgery, Massachusetts General Hospital, Boston, MA (Dr. Yellin, Dr. Lu, Ms. Duane, Dr. Berkson, Dr. Bono, Ms. Duffy, Dr. Fogel, and Dr. Zarins); the Department of Orthopaedic Surgery, Brigham and Women's Hospital, Boston, MA (Dr. Bluman, Dr. Ready, Dr. Weaver, and Dr. Dyer); the Department of Orthopaedic Surgery, Boston Children's Hospital, Boston, MA (Dr. Bauer and Dr. May); and the Department of Orthopaedic Surgery, Beth Israel Deaconess Medical Center, Boston, MA (Dr. Appleton and Dr. Drew).

## Abstract

**Introduction:**

When the COVID-19 pandemic forced the cancellation of visiting subinternships, we pivoted to create a virtual orthopaedic rotation (VOR). The purpose of this study was to assess the effect of the VOR on the residency selection process and determine the role of such a rotation in the future.

**Methods:**

A committee was convened to create a VOR to replace visiting orthopaedic rotations for medical students who are interested in pursuing a career in orthopaedic surgery. The VOR was reviewed and sanctioned by our medical school, but no academic credit was granted. We conducted three 3-week VOR sessions. During each session, virtual rotators participated in regularly scheduled educational conferences and attended an invitation-only daily conference in the evenings that was designed for a medical student audience. In addition, students were paired with faculty and resident mentors in a structured mentorship program. Students' orthopaedic knowledge was assessed using prerotation and postrotation tests.

**Results:**

From July to September 2020, 61 students from 37 distinct medical schools participated in the VOR. Notable improvements were observed in prerotation and postrotation orthopaedic knowledge test scores. In postrotation surveys, both students and faculty expressed high satisfaction with the curriculum but less certainty about how well they got to know each other. In the subsequent residency application cycle, 27.9% of the students who participated in the VOR were selected to interview, compared with 8.7% of the total application pool.

**Discussion:**

The VOR was a valuable substitute for in-person clinical rotations during the COVID-19 pandemic. Although not likely to be a replacement for conventional away rotations, the VOR is a possible adjunct to in-person clinical rotations in the future.

Orthopaedic surgery is one of the most competitive fields for medical students applying to residency.^1-3^ There are obvious challenges in scrutinizing hundreds of applicants each year to invite a fraction to interview and then ranking those applicants based on a series of 1-day interactions. To improve their chances of matching, medical students who are interested in pursuing a career in orthopaedic surgery typically participate in two or three 1-month-long clinical rotations at medical institutions other than their own during their last year of medical school. These “away rotations” afford the students the opportunity to not only become familiar with the field of orthopaedics but also to learn about a residency program and stand out among a growing sea of applicants as someone particularly interested in that program.

During a typical away rotation (or “subinternship”), potential applicants immerse themselves in the host program's culture. Residents and faculty have the opportunity to work closely with the rotator and note initiative(s) taken and preparation for the operating room and ability to work on a team. They observe the rotator's interactions with the nursing and operating room staff as well as with the resident team. These observations enable a program to gauge some of the intangible qualities of the applicant that cannot be learned from a written application. Reciprocally, rotators gain firsthand experience, working with residents and faculty and learning what distinguishes a particular program from others. Is this the right fit? Finally, it is well known that orthopaedic education is limited in medical school, and visiting rotations also serve to build an applicant's orthopaedic knowledge base and surgical skills.^2,3^

The COVID-19 pandemic forced the abrupt cancellation of nearly all in-person visiting orthopaedic clinical rotations for medical students in 2020. Facing the sudden loss of this opportunity, similar to how several programs adopted a virtual learning platform for *current* trainees during the pandemic, our residency program created one of very few formal medical student virtual orthopaedic rotations (VORs) in the United States.^4-6^ In creating a structured VOR, our goal was to replicate the three goals of a typical visiting rotation as effectively as possible in a virtual setting:

1. For our faculty and residents to “meet” and assess VOR participants.
2. For VOR participants to “meet” and assess our residents, faculty, hospitals, and educational program.
3. To supplement the orthopaedic education of VOR participants.

This study reports on our design process and content of the rotation. In addition, we aimed to evaluate the results of the VOR on our residency selection process in an attempt to understand whether the VOR would be worthwhile to continue after the current pandemic is over.

## Methods and Virtual Orthopaedic Rotation Design Process

For this prospective observational study, we convened a volunteer Virtual Rotation Education Committee to craft, refine, and run the VOR. The committee comprised 16 faculty members and residents and met virtually on a weekly basis before, during, and after the VOR. Faculty committee members were responsible for creating the VOR curriculum and recruiting faculty speakers and mentors. Resident committee members were responsible for recruiting resident mentors and publicizing the rotation to potential applicants.

The committee addressed practical concerns presented to us by our medical school and hospitals. During the pandemic, our medical school dictated a 3-week duration for all in-person clerkships in our medical centers, and we elected to mirror that for the VOR. Our plan to offer a VOR was reviewed and approved by our medical school registrar, but no tuition was charged and no academic credit was offered. We were instructed by the registrar's office to limit participation in the VOR to students who were enrolled in US medical schools. Our hospitals' education and privacy offices reviewed the Health Insurance Portability and Accountability Act implications and onboarding requirements for the virtual rotators. To comply with the Health Insurance Portability and Accountability Act, we carefully scrubbed any patient-specific information from the material that would be shared with VOR participants. Because virtual rotators would have no contact with patients and no exposure to protected health information, no formal onboarding was required.

A simple online application was created to elicit basic demographic information, a curriculum vitae, and a short free-response question asking the applicant to state why they wanted to participate in the VOR program. The application was disseminated through our residency program's social media channels and our residency program's website. Our program coordinators also distributed it by e-mail directly to students who expressed an interest in our program through a virtual information session hosted by our medical school or by contacting the residency office. All applicants who demonstrated enrollment in US medical schools were accepted into the VOR program.

The backbone of the VOR schedule was a series of 1-hour evening lectures and discussions led by faculty members. These were designed specifically for a medical student level of education. Faculty-led sessions met by Zoom each evening from Monday through Thursday. The Friday evening session was reserved for resident-led sessions.

Curricula for the evening lecture series were designed by the Virtual Rotation Education Committee and covered a wide array of orthopaedic topics. Sessions varied in structure, ranging from instructors assigning prereading and then pursuing a flipped-classroom model to a more standard lecture format with interactive components throughout. The Friday evening sessions were led exclusively by residents and were designed for the applicants to get to know the current residents and vice versa. These evenings had loose themes, including “Living in Our City,” “The Academic Curriculum,” and “Navigating Our Academic Medical Centers” (Table 1).

**Table 1:** Sample Evening VOR Schedule

	Monday	Tuesday	Wednesday	Thursday	Friday
Week 1	Anatomy/Orthopaedic History and Physical Examination	Foot and Ankle: Tendon Transfers of the Lower Extremity	Pediatrics: Clubfoot & Scoliosis	Trauma: Hip Fractures *or* Initial Management of Extremities	Resident Social: “Living in Our City”
Week 2	Spine: Spine Anatomy	Oncology: Introduction to orthopaedic Oncology	Pediatrics/Trauma: Common Pediatric Injuries	Arthroplasty: Total Knee Arthroplasty—the basics	Resident Social: “The Academic Curriculum”
Week 3	Sports: ACL Injuries	Hand: Distal Radius Fractures	Pediatrics: Developmental Dysplasia of the Hip (DDH)	Sports: Young Patients with Hip Pain	Resident Social: “Navigating Our Academic Medical Centers”

VOR = virtual orthopaedic rotation

Sample Evening VOR Schedule—An outline of the dedicated hour-long evening session topics for the medical students participating in the 3-week VOR. Each topic was led by different participating faculty members.

Questions and discussions were encouraged with the overarching goal of delivering answers that the virtual rotators would have gleaned had they been rotating in-person. Smaller virtual groups were also created to have more intimate conversations and get to know the rotators on a more personal level because the 3-week sessions varied in size from 11 to 29 students.

VOR participants were invited to participate in our program's weekly resident-wide Comprehensive Orthopaedic Resident Education curriculum on Wednesday mornings and were also presented with a comprehensive list of regularly scheduled daily conferences in each orthopaedic subspecialty division. The latter was optional, and virtual rotators were excused if they were involved in their own home rotation during the daytime.

Furthermore, VOR participants were paired with both a resident and faculty mentor immediately preceding their rotation. Both mentors were responsible for at least two to three “check-ins” throughout the 3 weeks and served as a resource throughout the rotation. This was another way to establish enhanced personal connections for both the mentor and the mentee. Although not required, many mentors continued to be engaged with their mentees after the rotation and throughout the application process.

Of note, this study was determined through a departmental process to be a quality improvement project and not research requiring an IRB.

## Results

### Demographic Reach of VOR

From July to September 2020, 61 students participated in the VOR. We ran three 3-week sessions with similar curricula. Eleven students participated in the first session, 21 in the second, and 29 in the third.

Overall, our virtual rotation allowed us to engage with more students from outside institutions than in the past. We had 61 virtual rotators compared with 41 in-person rotators in the previous year. This corresponded to a 48.8% increase in participation. In addition, there were increases in the number of female participants, total medical schools, and total states represented. No difference was observed in the number of underrepresented minorities who participated between the virtual rotation this year and the in-person rotation last year (Table 2).

**Table 2:** Demographic Reach of the Virtual Orthopaedic Rotation

	Academic Year 2019 to 2020 Sub-interns	Academic Year 2020 to 2021 Virtual Rotators	
Total # of students	41	61	48.8% Increase
#of Schools represented	32	37	15.6% Increase
#Female	13	20	53.8% Increase
#URM	12	12	No change
#States represented	18	22	22.2% Increase

URM = underrepresented minorities

In the 2020 to 2021 application season, we received 733 qualified applications for our 12 residency match positions. We invited 64 applicants to interview, 8.7% overall. Of these, 17 applicants (26.6%) had participated in the VOR. Of all VOR participants, 27.9% (17 of 61) were invited to interview. Furthermore, of these 17 interviewees, eight (47%) were ultimately ranked within the *matchable range* at our institution in the 2021 match. Of these eight applicants, six matched at our institution and matriculated as residents.

### Orthopaedic Knowledge Assessment

**Figure 1:**
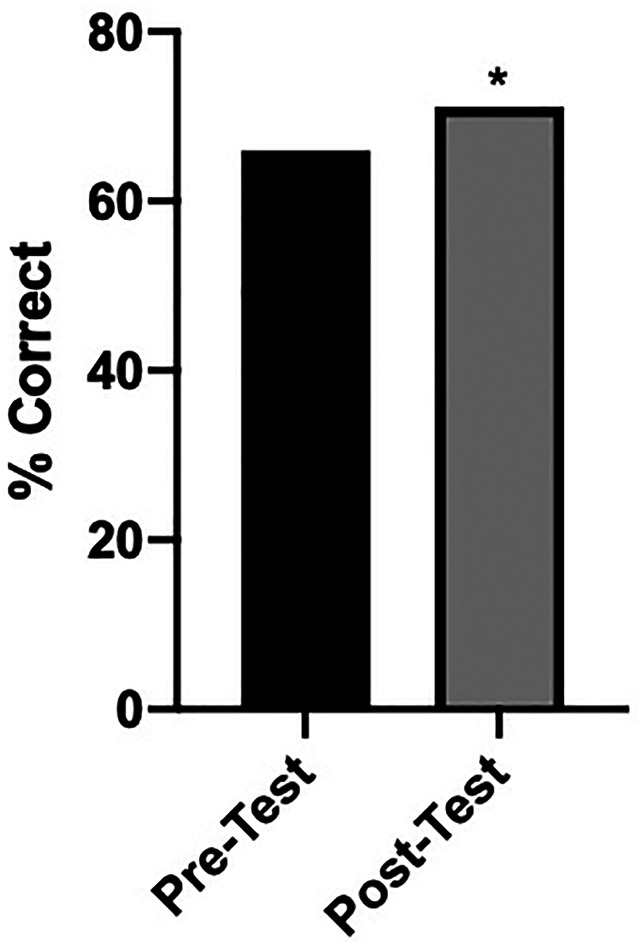
Graph showing pre-VOR and post-VOR test scores—students participating in the VOR were administered a prerotation and postrotation JBJS Clinical Classroom examination covering a wide array of orthopaedic topics. This shows the aggregate of all pre-VOR and post-VOR test scores compared using the Student *t*-test. *Statistically significant (*P* < 0.05). VOR = virtual orthopaedic rotation

Students were asked to complete prerotation and postrotation tests to assess their orthopaedic knowledge before and after the VOR. Fifty-eight of the students completed both tests. Each test consisted of 18 questions which spanned Ethics, Trauma, Sports Medicine, Hand and Wrist, Pediatrics, Adult Hip Reconstruction, Adult Knee Reconstruction, Spine, and Foot and Ankle. The tests were administered through the JBJS Clinical Classroom. There was a statistically significant increase in pretest and posttest scores from a mean of 66.0% ± 12.0 to 71.2% ± 8.5 (*P* = 0.0081) (Figure 1).

### Participant Surveys

We surveyed VOR participants at the end of each session and surveyed faculty and residents at the conclusion of the program.

The student survey contained two free-response questions: (1) What was the most valuable feature of the rotation? (2) What is one thing we should change to improve the rotation? Fifty-two students (85%) responded to the survey. Thirty-six (69.2%) felt the most valuable aspects of the VOR was interacting and connecting with current residents. Twenty-nine students (56%) mentioned meeting and interacting with faculty. Twenty rotators (38%) specifically mentioned their appreciation for working on a one-to-one setting with their mentors. Nineteen students (36%) highlighted the VOR curriculum itself, including the dedicated evening lectures. (In answering the first free-response question, many students mentioned more than one single feature, thus the percentages total more than 100%.) Overall, students emphasized the benefit of interacting with faculty and residents and expressed that they had a better understanding of the program after they had completed the VOR. One participant stated, “After the virtual away rotation, I feel like I have a great idea of what the XXX Orthopaedic Residency Program community is like and the dynamic of the learning environment.”

Although there was a great deal of positive feedback, students also shared suggestions for improvement. These comments were generally more specific, and rotators typically limited their responses to one recommendation. In order of prevalence, students expressed desires for (1) smaller group sessions (23%)—particularly among the session 3 students, (2) more interactive lectures and/or with associated prelecture assignments (14%), (3) more interaction with current residents (12%), (4) a greater emphasis on student-student interaction (10%), and (5) greater clarity with daily conference scheduling (10%). A few students expressed interest in more opportunities to showcase their knowledge, such as requiring student presentations at the end of the rotation. Twelve rotators (23%) expressed great satisfaction with the VOR, as is, with no additional recommendations.

We also surveyed the faculty and residents who were involved in the VOR. Of the 21 faculty and residents who responded to the survey, 18 (90%) either responded “yes” or “maybe” to continuing the virtual rotation in the future. The overall sentiment was that in-person rotations are still the best tools for our program and students to get to know each other but that the virtual rotation was a good alternative during an unprecedented application cycle. Those who recommended offering the VOR in the future cited increasing the opportunity for students throughout the country to interact with our program, particularly for students who would not be able to participate in an in-person rotation for logistical or financial reasons.

Finally, we encouraged the six residents who participated in the VOR and ultimately matched at our program to complete a one-question anonymous survey (after matriculation) to determine how influential the VOR was in their ranking decision, by indicating “strong influence,” “moderate influence,” or “little-to-no influence.” Although one resident replied “little to no influence—I would have ranked this program at the top of my list regardless,” of the residents who completed the survey, 80% (four of the five residents) responded that the VOR had a “moderate influence” on ranking the program highly.

## Discussion

The orthopaedic away rotation is a crucial part of the residency application process, valued by applicants and programs alike. Because the COVID-19 pandemic forced the unprecedented cancellation of these clinical rotations, we created a VOR as a substitute. Over a 9-week period, 61 students from 37 medical institutions participated in this VOR. We found that the participants gained a statistically significant increase in their orthopaedic knowledge and improved the likelihood of receiving an invitation to interview at our residency program.

Participant surveys demonstrated that the applicants, faculty, and residents saw the VOR as beneficial but far from a perfect substitution for an in-person audition rotation. Some predictable limitations were unavoidable. From the resident and faculty perspective, it was difficult to evaluate individual applicant work-ethic and team integration, that is, the “behind-the-scenes” events and conversations that transpire during an away rotation. Similarly, the virtual rotators did not have the opportunity to see resident-resident interactions, such as in the resident work room, or faculty-resident interactions in and out of the operating room. On a logistical level, scheduling the hour-long evening sessions required some consideration because students were “logging in” from various time zones throughout the country, and several students continued to have clinical responsibilities at their home institutions.

Anticipating some of these concerns, we built adaptive innovations into the VOR. For example, pairing each rotator with both a faculty and resident mentor enabled each student to have consistent contacts over the course of the 3 weeks. Based on the feedback surveys, although inherently subject to bias among the students who were actively applying to the program, the rotators expressed clear appreciation for the dedication of the residents and faculty to the education of the rotators, success of the VOR, and commitment to ensuring each rotator had all questions answered. From the program's perspective, although unable to observe rotators in the OR or alongside residents in the Emergency Department or on the floor, faculty and residents were able to apprise the residency selection committee about insights they gleaned from their interactions with VOR participants, especially if they were part of one of the mentor-mentee partnerships.

Offering virtual rotations also produced some unanticipated benefits. Most of the medical students did not have to compromise full participation in their home program to simultaneously successfully complete the virtual rotation. Another obvious advantage compared with conventional away rotations was that any interested applicant accepted to our VOR, regardless of socioeconomic background or financial constraints, had much less financial burden for travel, lodging, and food. Previous research has already demonstrated that the orthopaedic residency interview process is a substantial cost for applicants that affects their decisions.^7^ In addition, conflicting away rotation schedules have historically forced medical students to choose to rotate at one program over another. In the future, virtual rotations would allow prospective applicants to participate in more than the customary 2 to 3 away rotations.

Interestingly, although not the only goal of the VOR, we found that overall the students improved their orthopaedic knowledge, as measured by pre-VOR and post-VOR tests. Beyond evaluating the “intangibles,” such as personality, fit, and work ethic, our rotation may have played a role in improving the orthopaedic education of our participating students.

For the Virtual Curriculum Faculty, beyond the initial work to design an interactive lecture/discussion, ultimately very little additional effort was required to revisit the curriculum in subsequent sessions. Furthermore, we were able to develop an online repository of accessible medical student level orthopaedic education material for future use in our program. This open access knowledge resource can also be shared in medical curricula that do not routinely teach or emphasize musculoskeletal topics. Moreover, our VOR program can serve as a blueprint for other programs looking to incorporate a virtual rotation in future years (in full or in part), ideally mitigating some of the notable upfront time and energy investment requirements.

The VOR program was inherently adaptable as well such that we could pivot and quickly respond to real-time feedback from applicants. For example, one early critique was that the rotators had limited ability to get to know one another. The following week, we changed the focus of the Resident-led Friday Night Session, breaking into smaller groups with several additional residents.

In the face of the global pandemic, we designed a virtual method to allow prospective residents to immerse themselves in our program. This was an opportunity for applicants to meet us, us to meet them, and to further their orthopaedic knowledge. Although not a replacement for prepandemic in-person rotations, based on feedback, the faculty, residents, and virtual rotators strongly encourage that elements of this program should continue in future years, even when restrictions are lifted.

The results from our surveys informed our plans for a follow-up on VOR in 2021 to 2022. With some in-person full-time clinical rotations permitted, we decided to hold a simplified version of the VOR during the summer of 2021 with the stated goals of providing some basic orthopaedic education and giving medical students an opportunity to learn about our program. This rotation included 8 weekly 1-hour evening sessions, comprising six didactic sessions and two virtual open houses. Each of the didactic sessions included faculty members from two different institutions who were encouraged to talk about our city and the program in addition to their clinical topic and to make the sessions as interactive as possible. Interested students could sign up for one or all lectures, covering a range of specialties, including a Q&A with current residents. These took place in the evening so that students on other clinical rotations could still attend. Approximately 40 students participated in each didactic session while the open houses drew over 100 participants. There was no application or screening process, no attendance requirement, and no assessments were involved.

In the event that further COVID-19 variants restrict in-person rotations, our program welcomes the opportunity to use the original VOR we developed. In the (hopeful) event that restrictions will continually gradually lift, our program plans to continue to take advantage of several aspects of VOR and adapt the VOR to the current environment as we have done this past summer (http://links.lww.com/JG9/A178).
